# How sexual and natural selection interact and shape the evolution of nests and nesting behaviour in fishes

**DOI:** 10.1098/rstb.2022.0139

**Published:** 2023-08-28

**Authors:** Ola Svensson, Charlotta Kvarnemo

**Affiliations:** ^1^ University of Borås, SE-501 90 Borås, Sweden; ^2^ Linnaeus Centre for Marine Evolutionary Biology, University of Gothenburg, SE-405 30 Gothenburg, Sweden; ^3^ University of Gothenburg, SE-405 30 Gothenburg, Sweden

**Keywords:** actinopterygii, female choice, mating competition, nesting resource, speciation

## Abstract

Among ray-finned fishes that provide parental care, many spawn in constructed nests, ranging from bowls, burrows and ridges to nests made of algae or bubbles. Because a nest by definition is a construction that enhances the nest-builder's fitness by helping it meet the needs of the developing offspring, nest-building behaviour is naturally selected, as is a preference for spawning with mates that provide well-built nests. However, nest-building behaviour can also be sexually selected, when nest traits increase mating success, protect against sperm competition or nest take-overs by conspecifics. Here, we offer a systematic review, with examples of how competition for sites and location of fish nests relates to sexual selection. We examine direct and indirect benefits of mate choice linked to nest traits, and different types of nests, from a sexual selection perspective. Nest-related behaviours are often under both natural and sexual selection, and we disentangle examples where that is the case, with special attention to females. We highlight some taxa in which nest building is likely to be sexually selected, but lack of research has left them uninvestigated. Some of them are established aquarium species, making them particularly amenable for future research. Finally, we compare with arthropods, amphibians and birds.

This article is part of the theme issue ‘The evolutionary ecology of nests: a cross-taxon approach’.

## 1. Introduction

A nest enhances a nest builder's fitness by helping it meet the needs of its developing offspring [1]. Nest-building behaviour, and choosiness for mates that provide well-built nests in good locations, are thus under natural selection. Importantly, however, nest-building behaviour can also be under sexual selection, creating an additional layer of selection on nest-building behaviour, which sometimes affects the shape and function of a nest, away from its naturally selected optimum. The distinction and interplay between natural and sexual selection in relation to nest building is a major focus of this paper.

We use the definition of sexual selection as ‘any selection that arises from fitness differences associated with nonrandom success in the competition for access to gametes for fertilization’ [2]. Sexual selection on nest-building behaviour can arise if the nest protects against sperm competition from sneaker males, or if one sex prefers to spawn in nests built in a certain way, and builders of attractive nests therefore gain increased access to opposite-sex gametes. Furthermore, when nest-sites are limiting and primarily (or only) site-owners gain access to gametes, then intra-sexual competition for nest-sites is also sexually selected.

In ray-finned fishes (Actinopterygii), it is mostly the male, alone or together with the female, that builds nests and care for the offspring [3,4]. Despite that pattern, males typically compete for mating opportunities, and females are choosy [5]. However, when preferred nests get filled with eggs, females may compete intra-sexually over those nests. This can generate both natural and sexual selection on female competitive ability and choosiness. We return to this topic in §7.

Fish nests (and bowers) have been thoroughly reviewed in an evolutionary ecology context previously [3,6]. Here, we build on this work and focus on aspects of nests that may be sexually selected and examine to what extent nest-holders and mate-choosers are under natural versus sexual selection. We have searched the literature within ray-finned fishes and the presented examples are meant to be systematically broad, illustrate points we want to make, and indicate areas where more research is needed. Box 1 describes our use of important definitions related to nests and eggs. The methods and the list of the reviewed literature are found in the electronic supplementary material 1.

Box 1. **Definitions related to nests and eggs.**

*Nests* in oviparous species are defined as constructions where eggs are laid and offspring are reared [1]. Nests should meet the needs of the developing offspring and are therefore by definition under natural selection [1]. If the construction has no function for rearing offspring, but it is solely used for mate attraction and spawning, it is not a nest, but a *bower* [7]. By *nest site* we mean the specific site where a nest is or will be built, whereas *nest* refers to a construction or modulation, carried out by the parent(s). Sites where spawning and care take place, but without any clear nest building are referred to as *breeding sites*. Although they are often referred to as nests or nest sites in the literature, strictly speaking they are not.

We define the eggs from a single female, spawned during a limited discrete period of time, as a *clutch*, and the fertilized eggs (and subsequent group of developing offspring) that together obtain parental care as a *brood*. A female may split its clutch into batches between nests and a brood may have several mothers and fathers, e.g. due to multiple females spawning in a male's nest and multiple males fertilizing those eggs, through reproductive parasitism. When females spawn continuously over an extended period of time, the line between different clutches will be obscured. Similarly, the line between different broods will be obscured when sequential and overlapping broods are cared for in the same nest.

Here we will show that (1) it is important to tease apart how natural and sexual selection act on nests and their builders, (2) different types of nests differ in how natural and sexual selection are likely to act on them, and (3) sexual selection can act directly on nest-related traits through various mechanisms before, during and after spawning.

## 2. Nest sites

### (a) Location and shape of nest sites

The location of a nest site is important for offspring survival and development and hence choice of site is naturally selected in both sexes. For example, in the three-spined stickleback, *Gasterosteus aculeatus* (Gasterosteidae), males prefer to build nests at sites with high oxygen levels or temperature promoting embryo development [8]. Moreover, the presence of predators induces change in male nest-site preference from open to more vegetated sites [9], and females choose males based on nest location, e.g. in shallow water [10] or concealed by plants [11].

There can also be sexual selection via competition over location *per se*, as in a lek. Large males of the Lake Tanganyika cichlid *Lamprologus callipterus* (Cichlidae) are central in the lek and females prefer to spawn with males surrounded by many neighbours [12]. Similarly, male sunfishes (Centrarchidae) dig bowl nests in densely packed colonies. In both northern sunfish, *Lepomis peltastes*, and the bluegill sunfish, *Lepomis macrochirus*, colonies are formed because unattractive males try to place their nests close to attractive males. The latter do not benefit from this arrangement, but do better when nesting solitarily to evade parasitic spawnings that are common within colonies [13–15]. By contrast, in the five-spotted wrasse, *Symphodus roissali* (Labridae), the edge of a breeding area is preferred by males as the location for their algal nest, presumably because such sites are more visible to females [16].

The cichlid *Julidochromis transcriptus* uses breeding sites in rocky crevasses. Females prefer to spawn in wedge-shaped crevasses over those with parallel sides, as the former shape prevents a larger male from expelling a smaller male from the narrow part. The female benefits from strategically spawning in such locations, because when two males gain paternity, the brood receives paternal care from both males, resulting in overall more brood care in terms of fanning and cleaning of the eggs and embryos. Female choice of breeding site is therefore under natural selection, since females invest less care while offspring receive more care in trios than when females are paired to single males. It also affects sexual selection among males, by decreasing the reproductive skew and hence the opportunity for sexual selection [17,18].

### (b) Competition over nest sites

Male competition for nest sites falls under natural selection, since the site is needed for the male to provide paternal care. It is *also* under sexual selection whenever owning a site is instrumental for a male in gaining access to female gametes.

Competition over nest sites is common among gobies, and it has been studied in unusual detail in the sand goby *Pomatoschistus minutus* and common goby *Pomatoschistus microps* (Gobiidae), as shown in box 2. While the sand and common gobies are nest-builders, the two-spotted goby, *Pomatoschistus flavescens,* does not build nests, but uses empty bivalve shells or kelp as substrate for egg-laying and care. In nature, selection on male body size in relation to breeding site competition varies over the season due to changes in adult sex ratio, from male- to female-biased [19], and experimentally aggregated breeding sites skew mating success towards large males by enabling them to monopolize several sites [20]. Among fluvial gobies (Gobiidae), such as *Padogobius bonelli, Rhinogobius* sp. ‘DA’, *Tridentiger brevispinis* and *Sicyopterus lagocephalus*, which spawn under stones, large males occupy large breeding sites, which can house many eggs [21–23]. Females have been shown to prefer to spawn in large breeding sites [21,23], thus enhancing sexual selection on males for holding such sites.

Plainfin midshipman, *Porichthys notatus* (Batrachoididae), offers another example of strong competition for nest sites. Males of this species excavate nests underneath rocks in the intertidal zone. Larger nests can accommodate larger broods, and males prefer larger nest sites, while females prefer larger males [24]. Nest take-overs by conspecifics are common, especially early in the season. However, male size and condition have surprisingly little effect on the likelihood of a male keeping or taking over a nest [25], in contrast to, for example, sand and common gobies (box 2).

Box 2. **Sand goby and common goby nests**

**Figure 1.**
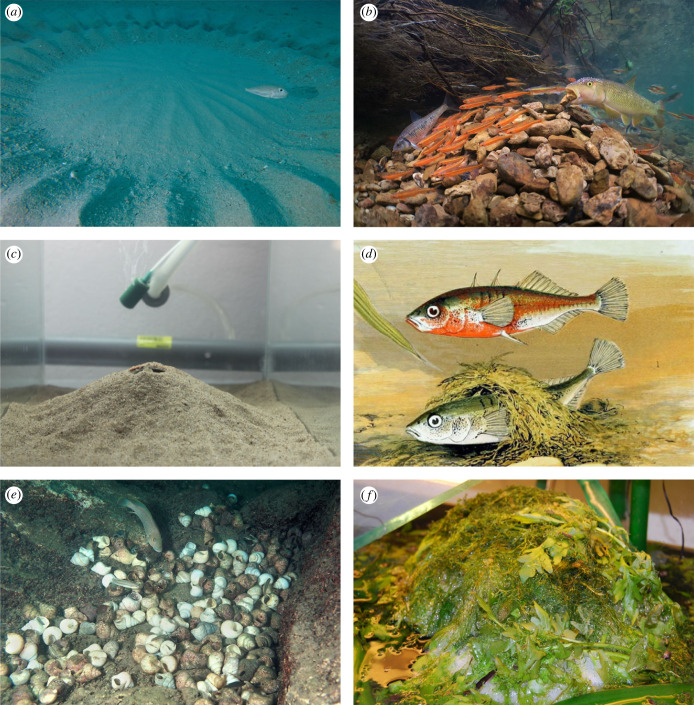
(*a*) Male white-spotted pufferfish *Torquigener albomaculosus* builds an elaborate construction of radial valleys and rings up to 2 m in diameter (photo credit: Hiroshi Kawase, Coastal Branch of Natural History Museum and Institute, Chiba). (*b*) Male redtail chub, *Nocomis effusus*, builds a dome nest of rocks. Other minnows that are associated with the nest spawn mutualistically at the nest and dilute the predation risk of both eggs and nest builders. Mutualistically spawning nest associates include striped shiner, *Luxilus chrysocephalus*, Tennessee shiner, *Notropis leuciodus,* and rosyside dace, *Clinostomus funduloides.* All these species belong to the Leuciscidae family (credit: Isaak Szabo). (*c*) Male sand goby, *Pomatoschistus minutus*, builds a nest by covering a nest site (e.g. bivalve shell in nature; halved flower pot here) with sand, forming a small entrance and preparing the inside with mucus (photo credit: Ola Svensson). (*d*) Excerpt of a painting from 1879 by A. F. Lydon (1836–1917) showing a nest of the three-spined stickleback, *Gasterosteus aculeatus*, built of algae held together by a glycoprotein produced in the kidneys. The male nest builder is above the nest and the spawning female in it (photo credit: Public domain). (*e*) Male *Lamprologus callipterus* builds a nest by collecting many empty *Neuthauma* shells. Females use the shells as breeding sites, while the male (too large to enter any shell) guards the nest. Another species of cichlid, *Telmatochromis vittatus*, collects no shells of its own but uses *L. callipterus* nests as breeding sites (photo credit: Ad Konings). (*f*) Male catfish *Hoplosternum littorale* builds a bubble nest of mucus-covered bubbles produced in the buccal cavity. His modified pectoral fins direct the bubbles to the pelvic fins, where they are whipped into a foam. The pectoral fins are also used to insert plant parts into the nest (photo credit: Joel Rahkonen). (*a–f*) For more information on behaviour and species identification within photographs (*b*) and (*d*), see electronic supplementary material 2 and electronic supplementary material, figure S1.

Sand goby, *Pomatoschistus minutus*, and common goby, *Pomatoschistus microps,* are nest builders that quickly occupy nest sites and build nests in both aquaria and in nature (figure 1). Their nest-building behaviour has been studied extensively, especially from a sexual selection perspective. To highlight this body of knowledge on both nest-site competition and sexually selected nest building, we dedicate this box to these two species. Both species reproduce in shallow sandy or muddy bays. The male excavates a burrow underneath the nest site, often an empty bivalve shell, covers the top by fanning sand or mud with its tail, and forms a small nest opening. He then attracts several females to spawn sequentially in this nest, before entering a 1–3 week parental care phase.

**Nest-site competition and sexual selection**

To build a nest, the male needs a nest site. If site availability is limiting in terms of numbers or sizes, this can affect sexual selection:

(a) *Nest-site numbers*: A shortage of nest sites can induce competition among males, and also competition among females for nest-holding males with space for them to spawn their eggs [26–29].
(b) *Nest-site size*: A holder of a large nest site can get greater reproductive success because more eggs can fit inside it [30]. Experimentally, large sites (especially when combined with few sites, or with females spawning asynchronously) lead to higher opportunity for sexual selection among sand goby males [29,31]. Natural habitat (small shells in sand versus large round rocks without sand) also affects sexual selection [32]. Large nests are coveted by conspecific males, and under site limitation, large nests get gradually taken over by larger males [30]. Female preference for large nest sites is found in common gobies [33] but not in sand gobies, e.g. [34]. Still, nest-site size affects paternal care and male courtship, which in turn females pay attention to in their choice [35].
(c) *Male size and condition*: The size and condition of the male affect his resource holding potential, that is, his ability to acquire and hold on to a nest site [30,36,37]. Large males are preferred by females [38,39], especially when holding a large nest site [34].

**Nest building and sexual selection**

Male nest-building behaviour before spawning is under sexual selection because it affects the male's ability to attract females, and to defend his paternity:

(a) *Mate attraction*: Both sand and common goby females prefer to spawn in well-built nests that are well covered and/or have a small nest opening [40–43] (but see [44,45]). These nests are typical for males in good condition [44,46–48], and may indicate e.g. low rates of filial cannibalism [48]. Males emit a courtship sound, which is important in female choice [47], and a thick layer of sand on top of the nest amplifies the courtship sound [49]. Consistent with this, female spawning decisions correlate positively with how much sand is placed on top of the nest [41,43].
(b) *Parentage*: Parasitic fertilizations are common, both by sneaker males (small males without nests) and nest owners [26,45,50,51]. Sand goby males build small nest openings in the presence of sneaker males, presumably as a defence against sneak intrusions [52] (but see [45]). Goby males prepare their nests with a sperm-containing mucus before spawning [53], and sand gobies spend more effort on mucus preparation when housed together with sneaker males than when alone [43].

In trout and salmon (Salmoniidae), females build depressions in the gravel—so-called redds, where the eggs are laid—and they also compete for specific nest sites. In the brook trout, *Salvelinus fontinalis*, nest sites are reused and quickly occupied by other females when females are removed, suggesting naturally selected female–female competition over nest sites [54]. In brown trout, *Salmo trutta*, females build nests close to preferred males [55] and in chum salmon, *Oncorhynchus keta*, females are faster at constructing a nest when courted by preferred males [56]. We will discuss these examples further in §7a.

In socially monogamous fish, competition over breeding sites can be biased towards one sex. In the convict cichlid, *Amatitlania siquia* (Cichlidae), pairs are size dimorphic (males being larger) and compete over holes as breeding sites [57]. In an experimental study on wild fish carried out in nature, females were larger in areas of low than in high breeding site density, whereas male size was unaffected. Because sex-specific predation could be ruled out, the most likely explanation for this pattern is stronger competition over breeding sites among females than males [58]. By contrast, male smallmouth bass, *Micropterus dolomieui* (Centrarchidae) build bowl nests and provide excusive paternal care [59,60]. Smallmouth bass are predominately monogamous and sexual selection on males arises due to many unmated males, both with and without nests [60].

Nest-site shortage can create a situation where many males cannot find a nest site of their own, and they therefore resort to spawning parasitically. For example, in the peacock blenny, *Salaria pavo* (Blenniidae), a much larger proportion of sneaker males were found in a nest-site limited population, compared to a population with high nest-site abundance [61]. Similarly, in the fathead minnow, *Pimephales promelas* (Leuciscidae), nest-holding males guarded broods with lower paternity when access to nesting substrate was limited than when unlimited [62]. However, comparing a population of sand gobies with nest-site shortage to one with nest-site abundance, the proportion of paternity was remarkably similar [26,50], likely because not only sneaker males spawn parasitically, but also nest-holding males do so [26].

## 3. Benefits of mate choice based on nests

What benefits do choosers gain by basing their mate choice on nest traits? First, the nest *per se* provides direct benefits e.g. by protecting the offspring. Second, the nest is an extended phenotype of its builder, i.e. the part of the phenotype that is expressed beyond the body [63,64]. As such, it may carry heritable and non-heritable information about the nest builder [63]. These benefits may be both direct and indirect. Here, we use the well-studied sticklebacks (figure 1) as examples to illustrate the potential benefits that mate choice nest traits might provide.

### (a) Direct benefits of mate choice based on nests

Many direct benefits of nest choice derive from nests being naturally selected. The shape and location of the nest can protect the offspring from abiotic and biotic environmental factors [3,6,65–68]. Nests are typically also the place where the parents provide care [3,6]. As such, choice of nest site and nest construction is important to parents and offspring alike.

In fifteen-spined sticklebacks, *Spinachia spinachia*, females prefer to spawn in nests built high up in algae, and such nests are safer from egg predation [69]. In three-spined sticklebacks, males build looser nests in low compared to high dissolved oxygen, and females prefer to spawn in nest-designs that match the local environment [70]. Males in high condition build nests faster and more neatly [71], and males with higher cognitive abilities build more elaborate nests [72]. Thus, if care is linked to condition or cognitive ability of the parent, then nest appearance may carry information about future parental care.

### (b) Indirect benefits of mate choice based on nests

Indirect benefits of nest choice arise through offspring inheriting fitness-enhancing genes. First, if the nest is an indicator of the nest builder's ‘good’ genes—say if condition or cognition are heritable and correlate with nest traits—the offspring will inherit these genes. Second, when the propensity of building an attractive nest is heritable, the benefit is the propensity *per se*. When the preference for such nests is also heritable, Fisherian runaway selection may result in exaggerated nest building.

In three-spined sticklebacks, several studies hint at indirect benefits of female nest choice. Nest traits are repeatable within males and hence potentially heritable [73]. As mentioned, cognitive abilities and condition correlate with nest traits in this species [71,72], which therefore potentially indicate ‘good’ genes. Indeed, females with medium-to-low cognitive ability prefer males with high ability, whereas females with high cognitive ability show no preference, suggesting greater benefits for females with medium-to-low than high cognitive abilities [72]. If correct, these benefits are likely indirect. Another example is nests decorated with conspicuous natural or artificial material (such as coloured threads and foil sticks) and female preference for such nests [74,75]. Since the decoration itself has no obvious benefit to the offspring, it appears likely to function as an ornament. The decoration may advertise paternal skills [75], but indirect benefits are also feasible e.g. by Fisherian runaway selection.

A nest is constrained by its function of aiding the young, complicating the task of deciphering which traits are chosen and why. In the white stickleback, *Gasterosteus* sp., however, males build nest-like structures suspended in algae above the substrate [76]. By contrast to other sticklebacks, the male picks up the eggs after spawning and distributes them among the algae nearby and provides no further care [77]. Thus, the structure serves as a spawning site, but not as a nest where care is provided. Presumably, the function of the structure is to attract females and provide a place to fertilize the eggs, but whether it has any indirect benefits to the female is (as far as we know) unknown. Therefore, they resemble maternal mouthbrooding cichlids, in which males build bowers that are exclusively used to attract females and to act as spawning sites [7,78]. Suggested indirect benefits of bower choice include Fisherian runaway selection [79] and indicators of ‘good’ genes related to condition [80] and immune system [81]. Still, the benefits to females of bower choice are not fully understood even in these fishes. Shape, function and selection vary among species' bowers [78,82,83] and direct benefits of bower choice related to e.g. fertilization [83], predation [84,85] and filial cannibalism [85] may be present in some species. Male dominance hierarchies [79,84] and sperm competition [85] may also influence the selection of bower shapes.

## 4. Nest types and nest building relevant for sexual selection

Nest types can be organized into several categories, based on building material and shape of the nest construction. Here we review a few illustrative examples in each category, with the aim to point out cases where nest shape is known, or likely, to be under sexual selection, in addition to the omnipresent natural selection. In general, whenever there is sexual selection on nest-building behaviour, for example due to a preference for certain nest traits by the opposite sex, one would predict nest building to become more elaborate than it would be if the nest only served to protect the offspring from peril.

### (a) Elaborate nests: geometric circles, mounds and ridges

Some species build large and conspicuous geometric circles, mounds or ridges, requiring large investments from the builder. Intuitively, their shapes suggest functions beyond or in addition to that required to meet the needs of the developing offspring. For example, the 12 cm male of the white-spotted pufferfish, *Torquigener albomaculosus* (Tetraodontidae), builds a 2 m-wide structure consisting of circles and radial valleys in the sand (figure 1*a*) [86]. After spawning the structure gradually collapses. Initially, it was therefore assumed to have no function in paternal care, and hence considered a bower [86]. However, later research found that eggs are laid in the structure, and that the male cares for the eggs and maintains the structure [87]. Female choice based on these structures may be linked to both direct and indirect benefits. Hence, although their form presumably is a result of sexual selection on males competing over female gametes, structure shape and location may benefit the developing eggs and thus be under natural selection as well.

In some species of stream-living minnows (Leuciscidae), males construct mounds or ridges that are clearly nests that are designed to meet the needs of the developing offspring, as the male covers the spawned eggs with gravel [88]. Other species of minnows, referred to as nest associates, often join the nest builders and spawn at such nests (figure 1*b*). Because they dilute the predation risk of both eggs and nest builders, they are considered mutualistic nest associates [88]. In the bluehead chub, *Nocomis leptocephalus*, males excavate a large pit, fill it with gravel and continue to pile gravel into a 1 m-wide mound nest. Several conspecific males may co-breed on the same mound and defend and spawn in small separate pits on top of the mound [88–90]. Although some males build nests alone, co-breeding leads to larger mounds and higher mating success [89,90]. A potential direct benefit of co-breeding for both sexes is that eggs from conspecifics dilute the predation risk [91]. Hypothetically, a large nest may also attract more nest associates, further diluting the predation risk. Other examples of female preferences linked to nest building are found in this family. In the fallfish, *Semotilus corporalis*, sequential spawning with multiple females occurs in a nest that gradually forms a ridge of stones, and the male guards the eggs from predators [88]. Stone carrying and dropping are crucial parts of the male's effort to attract females and when a male drops stones into the nest, females have been described to rush into the nest for spawning [92]. Finally, in cutlip minnows, *Exoglossum maxillingua*, males carefully select the stones to be darker and more colour-saturated than the background when building their mounds [93], similar to sticklebacks that use algae of contrasting colour to mark the nest opening (§3b). It seems likely that choice of stone colour makes males more attractive to females, but as far as we know, this has not yet been investigated.

### (b) Burrows: digging and covering

Nests are commonly shaped as burrows, for example in gobies (Gobiidae), jawfishes (Opistognathidae) and catfishes (Siluriformes) [3], and are constructed by digging in the substrate often under an object. Additional nest building can include covering the object and stabilizing the entrance (e.g. by mixing mucus into the sand) [3].

A burrow with a narrow entrance is likely to protect eggs from predation, but curb ventilation. In sand gobies, during hypoxia males solve this trade-off by keeping a wide opening and attacking egg predators more frequently [94]. In addition to such naturally selected aspects of nest burrows, sexually selected nest-building behaviour is evident in sand and common gobies (box 2). Furthermore, in the blennid *Rhabdoblennius nitidus*, a nest site with a narrow opening enables the male force the female to spawn by preventing her from escaping [95].

Jawfishes are paternally caring burrow dwellers that dig extensive burrows, sometimes with the added support of shells, or vaulted stone chambers with tunnels to the surface [96]. Since the males are mouth brooders that live and brood within their burrows [97], the burrows are also nests. In the sequentially monogamous yellowhead jawfish, *Opistognathus aurifrons*, each individual from a pair occupies one of two single burrows that are spatially paired within a colony [97]. When, as often happens, individuals disappear from the area [97], these pairs of burrows open up for new rounds of mate choice. In the banded jawfish, *Opistognathus macrognathus,* unmated males may build a burrow next to a female's burrow and mate with her there. In other cases males and females form small groups of burrows, and observations suggest that females either spawn sequentially with the same male or alternate between males [97]. It is not known which sex, if any, is more choosy than the other [97]. There is, however, potential for mate choice based on burrow traits.

Among catfishes, madtom males, *Noturus* spp. (Ictaluridae), excavate crevices in gravel and provide paternal care to eggs spawned there [98–100]. The nest protects the offspring from predation and from being flushed away [99,100]. Evidence suggests that moving gravel in currents is energetically costly and that low-quality nests have reduced reproductive success [98]. In *Noturus exilis,* egg numbers correlate positively with male size and age, possibly because large and old males are preferred by females (sexual selection), or because large males build better nests or protect their eggs better (natural selection) [101]. In armoured catfishes (Loricariidae), *Pterygoplichthys* sp. [102] and *Hypostomus ternetzi* [103], males dig metre-deep burrows in riverbanks where they provide exclusive paternal care. Especially in *Pterygoplichthys* sp., an invasive fish established in many subtropical and tropical rivers, the nests show great variation in depth and width, and there can be thousands of burrows per km [102]. Although it has not yet been investigated, these huge mating arenas have high potential for studies on female choice based on nest characteristics; for example, male reproductive success could be correlated to nest shape and location.

### (c) Bowls: depressions in the substrate

Bowls are pits dug out in the substrate. They are a common type of nest, found in e.g. Centrarchidae, Characidae and Pomacentridae [3]. Bowls are often no more than shallow depressions [3], unlikely to be strong targets for sexual selection, apart from their location (§2a). That said, more intense digging requires energy [104], and some bowls are extensive constructions. In fact, many bowers (§3b) are architecturally bowls. Below, we provide a small sample of bowl-shaped nest constructions, likely to be at least partially under sexual selection.

Recently, a breeding colony estimated to contain 60 million active nests of icefish *Neopagetopsis ionah* (Channichthyidae), with male bowl nests (60 cm diameter) placed side by side, was discovered at approximately 500 m depth in the Weddell Sea, Antarctica [105]. Given paternal care and marked sexual dimorphism, with males being larger than females and having an elongated dorsal fin and lower jaw [106], this gigantic aggregation has potentially been shaped by sexual selection on male nest building. Little is known about the reproductive ecology of this species, but in other icefishes, males search for nest sites and build bowl nests with low ridges [65,107]. During the breeding season, the anal fin of males of one of these species, *Chionodraco hamatus*, develops club-like knobs covered by a thick epithelium used in nest building, and potentially also in mate attraction [107].

In the world's largest freshwater fish, the Amazonian *Arapaima gigas* (Osteoglossidae), which is thought to be socially monogamous [108,109], the pair digs a cylindrical, roughly half-metre wide hole, surrounded by a cleaned area several metres in diameter. After days of shared nest building, the pair spawns. The cleaned area and the hole are thought to make the eggs easier to protect against egg predators. The female leaves after hatching and the male continues to protect the fry and later to herd the young through flooded forests over a 3-month period [108]. However, genetic analyses show that broods cared for by males typically consist of clutches from more than one female [109]. Hence, the first biparentally built nest may be sexually selected in males by the spawning of additional female(s).

The bowfin, *Amia calva*, is the only nest-building ray-finned fish that is not a teleost. It was long considered to be the only species in the order Amiiformes, but in 2022 it was divided into two species [110]. Males build large bowl-shaped nests under logs, stumps or bushes, by removing plants, shoots and roots [111–113]. The number of nests in an area depends on the amount of suitable spawning territories [113]. Males attract females to the nest by courtship [111,113], and offer exclusive paternal care with fierce protection of the offspring [111–113]. Some species of minnows spawn as nest associates (cf. §4a and figure 1) in bowfin nests [114,115]. Bowfins are common and widespread top predators, but despite their ecological importance and commercial value in the flesh and roe market, our knowledge of the ecology of bowfin is scarce [116,117]. Apart from what is outlined here, evolutionary ecology and behavioural ecology studies on the bowfin itself are apparently lacking, thus also in relation to mate choice and nest building. These iconic and phylogenetically unique fish certainly need further investigation.

### (d) Plants and algae

Several species of sticklebacks build nests of algae held together by a glue secreted by the kidneys (§4f). However, other fishes also build nests out of plant material, without using glue. For example, *Symphodus ocellatus*, *Symphodus tinca* and *Symphodus melops* (Labridae) build nests of algae, sometimes mixed with sand or pebbles [118–120]. In *S. ocellatus* and *S. tinca*, female mate choice is based on several nest-related traits: females prefer to spawn in young nests, with many newly laid eggs and few parasitic males nearby [121,122]. In *S. melops*, nest size correlates positively with male size. In a ‘no-choice’ set-up that tested whether a single male's nest was acceptable as a spawning site, *S. melops* nests of all the sizes that were offered to females were accepted [120], indicating that nest size (along with multiple male-related traits) has limited importance for female choice in this species. Still, in nature several nests are likely to be evaluated simultaneously or in sequence. Therefore, this result would be more conclusive if each female were offered at least two nests of different sizes.

The freshwater elephantfishes (Mormyridae) also use plants as building material. They are able, even in darkness, to distinguish between shapes and materials using electrolocation [123]. *Pollimyrus isidori* and *Pollimyrus marianne* males build nurseries of plant material before courtship and spawning [124,125]. Yet, spawning does not take place inside the nursery but beside it and the male moves the eggs into it after spawning (i.e. not a nest *sensu stricto*). The male often builds several nurseries but uses only a subset of them, with multiple clutches from one or several females in each [124]. Whether any nursery-related traits affect spawning success is however unknown.

Some biparental Cichlasomatinae cichlids spawn on loose leaves [126–128]. Female *Andinoacara coeruleopunctatus* prefers stiff leaves, which appear to be a limited resource [126,127]. In a study in nature on a species that was misidentified but probably is *Krobia guianensis* (see electronic supplementary material 1), the female is described to grasp a leaf by her mouth and move it around during courtship [128]. After spawning, either parent may move the leaf to a spot that is safe from predation, or to deeper areas if water levels drop [126–128]. Again, it is unknown if being an owner of a leaf of the preferred quality affects spawning success of either sex.

### (e) Animal matter: snail shells

Several Lake Tanganyika cichlids build nests of empty *Neothauma* gastropod shells. *L. callipterus* males build a nest consisting of a large collection of shells [129] over which they compete (figure 1*e*) [130]. The male protects the nest and the female provides care, but the male is too large to enter the shells and performs no direct care [129]. Large males accumulate more shells than smaller males, thereby enabling and potentially attracting more females to breed in his nest [12,129]. Males also collect additional shells that are too small for females to use for breeding, suggesting an ornamental function of such shells [131] (but see [12]). Importantly, females do not appear to prefer large males or males in high condition *per se* [132], which means that male size increases reproductive success primarily via the size of the nest. Meanwhile, females compete over shells *within* a nest [130] and prefer to occupy large shells [131,132].

*Lamprologus multifasciatus* is a much smaller cichlid than *L. callipterus* and males build nests by uncovering *Neothauma* shells from the sediment. The shells provide shelter and breeding sites for females. As in *L. callipterus,* females compete over shells within a male's nest [133,134]. A female will change to another male's nest if that allows her to occupy more shells for reproduction [134]. Experimental addition of shells increases male attractiveness. While more females join such nests, they also carry costs in terms of increased aggression from neighbouring males and increased risk of being predated [135].

In signal blennies*, Emblemaria hypacanthus* (Chaenopsidae), males also use empty gastropod shells as breeding sites. At a natural site with large shells, male size was a good predictor of reproductive success. However, at another site with only small shells, shell size was a better predictor for reproductive success than male size, and there was low variance in reproductive success, since most males got their shells filled with eggs [136]. Thus, size of the breeding sites can clearly affect the opportunity for sexual selection among males (cf. *J. transcriptus*, §2a).

### (f) Self-produced nesting material: glue, mucus and bubbles

Some fishes produce nesting material themselves, and again, there is evidence of both natural and sexual selection behind these adaptations. The algal nests of sticklebacks and tube-snouts (Aulorhynchidae) are held together by a glycoprotein-based ‘glue’ produced in the kidneys (figure 1*d*) [137]. In sticklebacks, glue production is linked to the condition of the nest-building male [71,138], and females prefer to spawn in well-glued nests [138]. Thus, assuming that a well-glued nest benefits offspring survival, this female preference is naturally selected, while male ability to build it is both naturally and sexually selected. Furthermore, apart from the direct benefits accrued from the nest, a female may also gain phenotypic and genetic information about the male, based on the amount of glue in the nest.

A glycoprotein-based mucus is another nesting material produced by nest builders. The fringed darter, *Etheostoma crossopterum* (Leucosidae), produces a mucus with antimicrobial properties that increases egg viability. The mucus is excreted from a dorsal patch behind the head [139]. In Gobiidae, the nest-holding male also prepares the spawning substrate with mucus, excreted from their sperm duct glands (e.g. [140]), and similar to darters, the mucus contains antimicrobial compounds that keep the developing eggs healthy [67]. In addition, it contains pheromones [141], suggesting a function for mate choice, and it plays a role in sperm competition (§5a).

Bubble nests consist of a foam and are built of bubbles produced by the parent, although plant matter can be included as well (figure 1). Most bubble-nesting species have paternal care [142–144], and most, if not all, are air breeders associated with stagnant freshwater [142–144]. Unfortunately, direct tests of mate choice related to nest traits appear to be lacking, but it would be interesting to see this explored.

One of the more well-investigated families with bubble nests is gouramis (Osphronemidae). Their floating nests are built from bubbles produced in the buccal cavity, covered with glycoprotein mucus [111,145]. Unlike gobies and darters, this mucus does not appear to have antimicrobial functions [146]. In nests of male fighting fish, *Betta splendens*, collected from nature, male length and weight correlate with nest size. However, neither size of the male nor the nest correlate with the number of eggs [147], and females do not prefer larger males in an aquarium experiment [148]. Similarly, in domestic strains of the fighting fish and the paradise fish, *Macropodus opercularis*, nest size does not correlate with the number of eggs [149,150]. However, the laboratory studies cited here were no-choice set-ups (i.e. only testing if the nests were acceptable for spawning, without offering any alternatives), and in nature, nest size was measured after spawning when, due to the ephemeral nature of bubble nests, any ornamental function of the nest is replaced by paternal care. Thus, with relatively simple improvements to the study designs, e.g. to measure the nest before spawning and let females choose between at least two nests, it would be possible to investigate the potential for mate choice related to nest traits in these fish further.

Another family with bubble nests is the armoured catfishes (Callichthyidae). Males of the subfamily Callichthyinae are known to build floating bubble nests [111,142]. In the *Hoplosternum littorale* catfish, the male builds a bubble nest before initiating courtship (figure 1*f*). Bubbles covered in mucus are produced in the buccal cavity and, using specialized pectoral fins, they are directed to the pelvic fins where the bubbles are whipped into a foam. A lot of effort is thereafter put into inserting plant material into the bubble nest, again using the pectoral fins [142,151]. There is large variation in the size of these nests, and since several females can spawn in one nest but more than half of the nests do not get any eggs, this suggests that there is strong competition among males for spawning females. Together, this indicates a clear scope for sexual selection acting on nest characteristics in these fish. During courtship, there is further nest building and the female may also produce bubbles [142].

## 5. Sperm competition and sexual selection after fertilization

Sperm competition, mate desertion and infanticide that happen after mate choice and spawning [152] can all lead to nest-related sexual selection. Here we give a few examples of each.

### (a) Sperm competition

In externally fertilizing fish, sperm competition happens after gamete release, but before fertilization [153]. Among nest-building fishes, it is common that males spawn parasitically, making sure their sperm are present along with that of the nest-holder, competing to fertilize the eggs laid in the nest [152].

The location of the breeding site or nest site can affect parasitic spawnings. *Telmatochromis vittatus* defends piles of shells that are nests constructed by the heterospecific *L. callipterus* (figure 1*e*) [154]. Behavioural observations show that large *T. vittatus* males occupy isolated piles rather than large piles with many females and that parasitic spawnings by pirate intrusion decrease with distance from other piles of shells [154]. This means that the optimal nest location for *T. vittatus* males is the opposite to that of *L. callipterus* males, since the latter species prefers to be central in the lek and females prefer to spawn with males surrounded by many neighbours [12] (§2a).

The shape of a nest entrance may also affect parasitic spawnings. In sand gobies, males build sand burrows with small nest openings in the presence of sneaker males (box 2) [52] and in the Lake Tanganyika cichlid *Lamprologus lemairii,* paternity correlates negatively with nest-site opening width [155]. However, in species that remove rival male sperm by fanning [156], a small nest opening may make sperm removal less effective. In *L. callipterus* (§§2a and 4e), a genetically determined dwarf male strategy has evolved as an adaptation to both the shells and the large territorial males. By contrast to nest-holding males, these parasitically spawning males can enter shells. They reside further inside the shells than females, fertilize eggs parasitically, and are hard for territorial nest holding males to remove [157].

In gobies, the male prepares the spawning substrate with an antimicrobial mucus [67] (§4f). The same mucus can also promote the fertilization success of the nest holder, as it contains embedded sperm that activate gradually as the mucus dissolves. This is presumably an adaptation to secure fertilizations, especially in the face of sperm competition from parasitically spawning males (box 2) [158]. Presence of their own mucus has also been shown to increase sperm velocity and fertilization rate of the nest holder in some of the studied species (e.g. [159,160]).

### (b) After fertilization

Sexual selection can also occur after fertilization. In species with biparental care, the female may be deserted by the male for another female [127,161]. Such desertion can decrease offspring survival through increased predation [161]. In biparental leaf-spawning cichlids (§4d), females compete over males and the stiff leaves that are used in courtship and as nests [127,128]. An unmated female with a leaf needs a male's gametes to reproduce at all (i.e. sexual selection) and may hence potentially affect the reproductive success of an already-mated female by getting the male to desert her. We will discuss this example further in §7.

A male that aggressively takes over another male's nest [152] may consume the whole or parts of the replaced male's clutch (e.g. [162,163]). In *L. callipterus* (figure 1*e*), males may steal shells from neighbouring nests or take over nests. In both cases, females are aggressively expelled from the shells and eggs and fry are consumed [129,130]. Hence, there is male–male and male–female competition over shells, both of which result in infanticide, and an increase in reproductive success of the winning male.

In some species of fish, females prefer to spawn in nests that contain eggs, over empty nests [158,164–167]. Explanations for this female preference span from egg presence functioning as proof that the male is a capable parent, risk dilution (cf. nest associates, §4a), mate choice copying, to large broods being more valuable and hence receiving more care from the male. It thus means that after one female has spawned in a nest, its attractiveness increases markedly to other females.

## 6. Assortative mating and speciation

Assortative mating in relation to nest building has been studied in distinct pairs of morphs (often referred to as species) within the three-spined stickleback species complex (*Gasterosteus* spp.). In Canadian sticklebacks (Misty Lake), a difference in nest construction between ‘lake’ and ‘inlet’ morphs is genetically determined and might therefore contribute to reproductive isolation [168]. By the same token, on Iceland (Lake Thingvallavatn), one morph nests in lava caves, another in *Nitella* algae. A strong assortative mating, shown in experimental aquarium experiments, is possibly driven by morph specific nest structure [169]. Nesting location can also contribute to reproductive isolation. In sympatric as well as allopatric Scottish stickleback pairs, assortative mating is driven by preferences for nesting microhabitat, rather than mate choice [170]. Similarly, in sympatric Canadian sticklebacks (Paxton Lake) studied in enclosures in an outdoor pond, ‘limnetic’ males build nests in open habitat, whereas ‘benthic’ males build their nests in vegetation [171]. However, in a ‘no-choice’ set-up testing whether a single male was accepted as a mate, a mismatch between male and habitat did not affect the probability of female spawning, suggesting that habitat isolation may be weak [171]. And finally, white stickleback males build nest-like structures in algae above the bottom (§3a) whereas males of a congeneric sympatric three-spined stickleback, from which they are reproductively isolated, build nests on the bottom. Studies carried out both in nature and in aquaria suggest that nesting microhabitat as well as nest shape contribute to the observed complete assortative mating [76,172].

## 7. Discussion

When nest sites, nests and their nest-holders are limiting resources, the term ‘mating competition’ often contains aspects of both natural and sexual selection. When the nest is a resource needed for offspring survival, competition for access to nests falls under natural selection, while competition for access to gametes is a clear case of sexual selection. In reality, when both assets are simultaneously offered by the nest-holder, an effort to separate them might seem vain. Still, for the purpose of book keeping and better understanding, it can be valuable to do so, at least as an academic exercise. Here, because it is less trivial and often ignored in evolutionary ecology [173], we focus on females, basing our discussion on the definition of sexual selection by Shuker & Kvarnemo [2].

### (a) Female competition over access to nest sites

As mentioned in §2b, brook trout females build nests, called redds, by digging a pit in the gravel, and they choose specific sites to build them [54]. Similarly, in *A. coeruleopunctatus* (§4d and §5b) [126,127], females prefer stiff leaves, which they use in courtship and as nest sites. Both redds and stiff leaves are limited resources essential for reproduction that females compete over, resulting in natural selection on their competitive ability. However, if females vary in their access to males—and hence sperm—due to variation in female nest traits, then females will also be sexually selected (alongside the natural selection to have a nest in the first place) to make or have a particular kind of nest. In sockeye salmon, *Oncorhynchus nerka*, artificial diggings that mimic the nests constructed by females just before spawning attract large numbers of males [174]. It is therefore feasible that females compete for males by digging, as well as in terms of quality, size, and location of the nest. Yet, this will result in sexual selection on females only if males or sperm are limited and there is competition among females for them. By contrast, when brown trout females build nests close to preferred males [55] and chum salmon females construct their nests faster when courted by preferred males [56], it is the preferred male that is under sexual selection.

### (b) Female competition over access to males with nests

When males build nests, and *nests that are preferred by females* (§3) get filled with eggs, this can result in female–female competition over access to such nests as spawning sites. This competition will generate natural selection if access to spawning space in preferred nests provides e.g. safety for the offspring. In this situation, females may also compete over access to gametes for fertilization—or access to genes to be inherited by the offspring—generating sexual selection on females. Similarly, when nests get filled with eggs, this can also result in female-female competition because there is a general *shortage of nest sites* (e.g. [27,61,175,176]). Females then compete over the chance to reproduce at all, generating natural selection, and for gametes, which generates sexual selection on females.

### (c) Choosiness and preference for nest traits

In §3, we asked how females benefit from being choosy regarding nest traits, and whether direct or indirect benefits can be identified. But how should we label the selection on female choosiness or specific preference that arises from these benefits? Most nest traits that benefit the offspring directly (e.g. through improved survival or development) generate natural selection on female mate-choice (§3a). Direct benefits related to nest building that benefit the choosy female herself (e.g. a safe spawning site) also fall under natural selection.

Because nests are constructions, they are extended phenotypes of the nest builder [63,64]. If the nest indicates parental abilities, then female choosiness and preference for nest traits will again be naturally selected, and females may compete for access to such males. However, if the nest indicates ‘good’ genes or genes that make the offspring attractive (able to build attractive nests; §3b), then choosiness and preference of the female will be sexually selected, *as long as* there is competition over males or sperm (i.e. they are limiting in some way) and having a preference helps the female gain access to more or higher-quality gametes. If there is no competition, however, the preference is naturally selected, since producing fit offspring is otherwise naturally selected.

### (d) Sexual selection in nest-building frogs, arthropods and birds

The overlap between natural and sexual selection in connection to nest-building behaviour reviewed in this paper is by no means unique to ray-finned fishes. Some frogs build foam nests. Their foam nests resemble the foam nests of gouramis and Callichthyinae catfishes (i.e. bubble nests, §4f) [111,142,143]. A visible frog foam nest increases a male's chances of mating [177] and nest building during mating arguably allows females to choose direct benefits through protection of the offspring from predation and desiccation [178]. Other types of male nests found among frogs are burrows [179] and water-filled bowls [180,181]. Although vocalization is a major part of male courtship, nest shape and location [177,179,180], as well as nest building *per se* [178], may be targets of female choice.

All examples mentioned so far are species with external fertilization. However, arthropods and birds with internal fertilization also offer good examples of nest building being the target of both natural and sexual selection. For example, in biparental Scarabaeidae dung beetles, brood balls out are made of dung before oviposition. In some species groups, the balls are made inside an extensive burrow (tunnel) where dung is transported, whereas in other species groups the male or the female instead rolls a brood ball above ground. In the latter group, the balls are used as sexual display and males compete over them and may steal balls from each other [182]. In *Scarabaeus catenatus* both the tunnelling and the rolling tactic exist. However, in both tactics, male participation appears to primarily function as mate guarding rather than parental effort [183]. Fiddler crabs (Crustacea: Ocypodidae) defend burrows in tidal zones. Males of some species build sand ornaments at the entrance of the burrows that increase their mating success but are washed away during the daily tide [184]. *Austruca annulipes* mates inside the male's burrow and the male leaves the burrow to the female for brooding. The female inspects several burrows before settling for a male and his burrow [185]. Male harvestmen *Quindina albomarginis* (Arachnida: Nomoclastidae) build bowl nests out of mud and perform exclusive paternal care. Females compete over males with nests and conspecific males may forcefully take over a nest and consume any eggs before attracting females [186]. Finally, in the burrow-building wolf spider *Allocosa senex* (Arachnida: Lycosidae) females choose males that construct long burrows [187]. A male will enlarge its burrow after female rejection [188]. After mating, the male leaves its burrow, seals its entrance and leaves the female caring for the offspring [187].

Birds are, however, the most studied group in this aspect. The variation in shape and construction techniques of their nests is immense [189] and, as shown in thorough reviews [190,191], it is increasingly clear that nests are also shaped by their functions in attracting and influencing the parental effort of mates [191]. Examples of the latter are found in blue tits, *Cyanistes caeruleus*, and great tits, *Parus majo*r (Paridae). The female builds the nest and incubates the eggs, whereas both sexes contribute to provisioning the nestlings. A high-quality female builds a tall nest inside a nest-box, which influences provisioning by the male positively [190]. However, this is not sexual selection, but natural selection. Across other species, correlational and experimental studies show that enhanced nest building, nest size or nest ornamentation increase attractiveness to females (i.e. sexual selection) and females may also increase care for nestlings in those nests [191] (i.e. natural selection). There is thus a clear concurrence of fish and bird nests, with nest building attracting mates (§3). However, we do not know of any example in fish where nest building has an effect on the mate's parental effort, as in blue tits and great tits, although it is feasible that it may occur in biparental nest-builders such as some cichlids [126] and catfishes [192,193]. Furthermore, the primary function of nest sealing in the black-and-white-casqued hornbill, *Bycanistes subcylindricus* (Bucerotidae), may be defence against nest take-overs by conspecifics [194]. There is thus male–male and male–female competition over these nest sites, both of which result in infanticide, with a clear resemblance to fish cases outlined in §5b.

### (e) Concluding remarks and future perspectives

The link between the nest's ornamental function and its function to meet the needs of the developing offspring is a likely reason why few, if any, confirmed cases are found of fish nests that have evolved to become more elaborate than required by natural selection. Based on observations, the white-spotted pufferfish (figure 1; §4a) may be an exception. Still, direct and indirect benefits of female choice based on nest traits have yet to be measured. The gradual collapse of the structure after spawning leads us to another important point: the nest must be measured when mate choice takes place. Nests are often ephemeral constructions in need of constant maintenance [46,86]. After spawning, any ornamental function disappears in most systems.

Studies suggest that the shape of the nest can defend against parasitic spawning and sperm competition (e.g. [52,195]). However, nest shape in relation to post-fertilization sexual selection, e.g. the nest opening protecting against nest take-over, is understudied, potentially because of its more obvious functions related to protection of offspring and carer.

Despite efforts, our review has a systematic bias towards cichlids, gobies and sticklebacks. There are many other groups of fish where too little is known or published to even speculate about sexual selection in relation to nests. However, other species are established in the aquarium hobby and have detailed descriptions in the non-scientific aquarium literature [196,197], but existing scientific studies are few or old. Examples include gouramis of the genera *Trichogaster* and *Trichopodus.* Another example is the catfish *H. littorale*, which is commonly bred in aquaria and aquaculture (figure 1, §4f) [142,198]. We wholeheartedly agree with Hostache and Mol's 25-year old statement that this species is a great candidate to study ‘female choice related to male characters like body size, colour and size of the pectoral spine, and size of the nest and territory’ [142, p. 182]. On a similar note, useful information about nest-building behaviour in fishes is often found in older studies (from more than 40 years ago) but lacking in more recent papers. Descriptive studies were common back then. However, many of these older studies are well grounded in theory and still have an admirable attention to details and additional observational information. What is needed to further increase our understanding of the diversity of nesting behaviour and sexual selection in fishes are studies that take advantage of recent theoretical and technical developments without compromising on detailed observational information.

Finally, in the very first paragraph of this review, we pointed out that when nest-building behaviour is under sexual selection, it may affect the shape and function of a nest away from its naturally selected optimum. To test that, one should manipulate nests to become more elaborated and (importantly) also less elaborated, and measure not only mating success but also offspring fitness. As far as we know, this has never been done, but should be on everyone's to-do-list.

To conclude, natural selection on offspring often (via mate choice) leads to sexual selection on parents, and there are plenty of behaviours related to nest building in fishes that are likely to be under sexual selection, in addition to the natural selection inherent in any construction called a nest. Basing our reasoning on strict definitions of what a nest is [1,7] and how to define sexual selection [2] has helped us elucidate, for example, that bowers and other breeding sites can be sexually selected without being nests, and that safe nests can generate natural selection on the sex that prefers it and both natural and sexual selection on the sex that provides it. We hope our review will inspire new research in this exciting area, especially within taxa that are known to have interesting nest-building behaviour, but that have not yet been investigated scientifically to their full potential.

## Data Availability

The list of the reviewed literature is found in the electronic supplementary material 1. The data are provided in electronic supplementary material [199].
